# Directional Confusions Reveal Divergent Inductive Biases Through Rate-Distortion Geometry in Human and Machine Vision

**Published:** 2026-04-23

**Authors:** Leyla Roksan Caglar, Pedro A.M. Mediano, Baihan Lin

**Affiliations:** 1 Windreich Department of AI and Human Health, Icahn School of Medicine at Mount Sinai, New York, NY; 2 Department of Computing, Imperial College London, London, UK

## Abstract

Humans and modern vision models can reach similar classification accuracy while making systematically different kinds of mistakes - differing not in how often they err, but in who gets mistaken for whom, and in which direction. We show that these directional confusions reveal distinct inductive biases that are invisible to accuracy alone. Using matched human and deep vision model responses on a natural-image categorization task under 12 perturbation types, we quantify asymmetry in confusion matrices and link it to generalization geometry through a rate–distortion (RD) framework, summarized by three geometric signatures (slope (β), curvature (κ)) and efficiency (AUC). We find that humans exhibit broad but weak asymmetries, whereas deep vision models show sparser, stronger directional collapses. Robustness training reduces global asymmetry but fails to recover the human-like breadth–strength profile of graded similarity. Mechanistic simulations further show that different asymmetry organizations shift the RD frontier in opposite directions, even when matched for performance. Together, these results position directional confusions and RD geometry as compact, interpretable signatures of inductive bias under distribution shift.

## 1 Introduction

Humans and modern artificial neural networks (ANNs) increasingly reach similar categorical decisions, but they often differ in the directionally biased errors they make. Most evaluation pipelines focus on performance accuracy and treat confusion as unstructured and aggregate-focused, asking how often a system errs, but not who gets mistaken for whom and in which direction (Gupta et al., 2021; Attarian et al., 2020). Yet, this directional structure is precisely where inductive biases leave their fingerprint. To uncover these differences, we leverage a well-established phenomenon in cognitive science, namely systematic asymmetries in visual perception and categorization. Perceptual asymmetries offer a lens into representational structure and inductive bias — the priors a system implicitly imposes when mapping ambiguous or degraded inputs to categories.

In cognitive science, human perception and categorization are known to exhibit robust asymmetries, showing that similarity is not a symmetric relation. People judge a robin to be more similar to a bird than a bird is to a robin, or an ellipse to be more similar to a circle than vice versa (Tversky & Gati, 1982). Such effects reflect structure in cognitive representations — including prototype effects (Rosch, 1975), feature salience (Tversky, 1977), category typicality (Rosch & Mervis, 1975), and hierarchical or causal priors (Sloman, 1998; Kemp & Tenenbaum, 2008) — that violate the first metric axiom and thus the assumptions of symmetric metric spaces (Shepard, 1964; 1987). Far from being mere noise, directional confusions in similarity judgments and identification/categorization tasks (Nosofsky, 1986; 1991; Getty et al., 1979; Kahana & Sekuler, 2002) can serve as diagnostic signatures of representational bias and efficiency (Sims, 2018; Jakob & Gershman, 2023).

In contrast, evaluations of ANNs under distribution shift typically abstract away error structure. Metrics such as accuracy or top-k error implicitly treat confusion patterns as symmetric or irrelevant (Geirhos et al., 2020; Recht et al., 2019). However, deep vision models can produce highly structured one-way failures. For example, classifiers may collapse diverse subcategories into a dominant prototype (e.g., many dog breeds into “Labrador”) without the reverse confusion occurring (Shankar et al., 2020; Miller et al., 2021; D’Amato et al., 2025). Under adversarial noise or texture-based corruptions, models may confuse texture-diagnostic categories (e.g., “zebra” with “barcode”) in one direction only (Geirhos et al., 2018a; Ilyas et al., 2019). Large-scale evaluations show that this gap between human and machine error consistency persists even in state-of-the-art models (Geirhos et al., 2021), and simplicity bias — the tendency of ANNs to rely on the simplest predictive features available — provides a mechanistic account of why such structured failures arise (Shah et al., 2020). These patterns motivate examining not only how large asymmetries are, but how they are organized — whether many pairs show small directional biases (distributed structure) or a few sink-like collapses concentrate failures into dominant categories. Recent work has compared human and machine confusion matrices at the level of aggregate error distributions (Liu et al., 2025), but without characterizing the directional organization of asymmetries or linking error structure to generalization geometry, which is the focus of the present work.

This raises a targeted question: When vision systems are matched for task, perturbation, and accuracy level, do humans and ANNs exhibit systematically different directional confusion structure? Cognitive accounts typically explain human asymmetries via graded similarity and attentional biases (Tversky, 1977; Rosch, 1975), suggesting broad but weak directional tendencies spread across many class pairs. By contrast, prototype collapse and shortcut-driven reliance on a small set of features in ANNs (Shankar et al., 2020; Geirhos et al., 2018b; Shah et al., 2020) predict sparser but stronger sink-like failures concentrated on a few classes. The representational geometry counterpart of this is a manifold collapsed onto a small set of dominant attractor states (Papyan et al., 2020), as opposed to the graded similarity gradients that characterize human-like representations (Sorscher et al., 2022; Wei & Woodford, 2025). These two organizations reflect fundamentally different priors. Distributed asymmetries suggest a system sensitive to graded similarity across many feature dimensions, while sink-like collapses suggest one that has learned a small set of dominant decision boundaries and applies them rigidly under shift. If this dissociation holds, it means two systems can show similar accuracy, or even similar aggregate asymmetry, while failing for qualitatively different reasons, with different implications for downstream reliability. We therefore not only compare asymmetry structure across systems, but explicitly test whether the organization of directional errors carries information about generalization that is independent of accuracy, by controlling for performance within matched blocks and asking what directional structure predicts above and beyond it.

To formalize how directional error structure relates to generalization, we adopt a rate–distortion (RD) framework grounded in information theory and efficient coding (Shannon, 1948; Sims, 2018). We treat each system — human or machine — as an effective communication channel defined by its stimulus–response confusion matrix, and summarize its generalization behavior as an information–error trade-off, asking how much mutual information must be preserved to achieve a given level of categorical distortion. Crucially, the RD framework accommodates asymmetric confusion matrices directly, without forcing the behavioral data into a symmetric similarity space, making it a natural formalism for comparing directional error structure across systems. We characterize the resulting trade-off using three geometric signatures of the inferred RD frontier: slope (β), capturing the marginal information cost of reducing expected error; curvature (κ), capturing how nonuniform this trade-off is across operating points; and efficiency (AUC). These signatures were introduced and validated in Caglar et al. (2026) as diagnostics of generalization geometry across biological and artificial visual systems. Within this formalism, the organization of asymmetry — not just its magnitude — predicts where a system sits on the RD frontier.

We address three linked questions. (1) Organization of directional confusions: do humans and ANNs differ in how asymmetry is organized — breadth vs. strength — beyond what accuracy captures? (2) RD geometry linkage: how does asymmetry organization relate to effective information–error trade-offs (AUC, β,κ), and is any coupling mediated by accuracy or does residual structure persist within matched performance blocks? (3) Robustness training: does robustness training reduce global asymmetry, and does it shift models toward the human breadth–strength profile? Our main findings, corresponding to each question, are as follows:

- We demonstrate that humans and ANNs dissociate in the organization of directional confusions — humans show broad, weak asymmetries while ANNs show sparse, strong directional collapses — revealing distinct inductive biases that are invisible to accuracy or global asymmetry magnitude alone.
- We link asymmetry organization to RD frontier geometry, showing that breadth–strength structure predicts efficiency (AUC) and shape descriptors (β,κ) above and beyond performance, with key associations persisting after controlling for accuracy within matched blocks.
- We show mechanistically that broad–weak and sink-like asymmetry regimes (corresponding to distributed vs. concentrated inductive biases) produce opposite effects on RD efficiency even with the same accuracy, formalizing why the same global asymmetry score can reflect fundamentally different generalization strategies.
- We show that robustness training reduces aggregate asymmetry toward the human range but fails to recover the human-like breadth–strength profile, highlighting the limits of scalar metrics for evaluating alignment.

## 2 Methods and Modeling Framework

### 2.1 Datasets, Perturbations, and Evaluated Systems

We analyze matched human and ANN model behavior on controlled perturbations of natural images in a K=16 ImageNet-derived categorization setting. The primary stimulus benchmark is the Generalization repository (henceforth GEN repository; (Geirhos et al., 2018b)), which includes twelve perturbation families (e.g., colour/grayscale, contrast, filtering, phase noise, power equalisation, rotation, Eidolon variants, and uniform noise), each parameterized by distortion strength to produce systematic out-of-distribution (OOD) conditions. The data includes ~83k human psychophysics trials, as well as three baseline pretrained convolutional neural networks (CNNs): GoogLeNet, ResNet-152, and VGG-19. To study training-induced variation, we evaluate models with different robustness regimes. In particular, we include (a) *Distortion-trained* ResNet-50 models trained from scratch with distorted training distributions, (b) *Specialised* single-distortion models trained on one perturbation family and evaluated across all perturbations, and (c) *All-noise / multi-corruption* models trained on mixtures of noise-like corruptions and evaluated across individual perturbations (see the GEN repository (Geirhos et al., 2018b) for further details on benchmark stimuli, model training, and task evaluations).

### 2.2 Confusion Matrices as Effective Behavioral Channels

We treat each system’s confusion matrix as defining an effective noisy channel, then ask what latent distortion structure — and what information–error trade-off — is implied by its pattern of errors, including their directional asymmetries. For each system s, experiment e, and distortion level d, we summarize stimulus–response behavior with a K×K confusion matrix $N^{\left(s,e,d\right)}$, where $N_{ij}$ counts responses of class j to stimuli of class i. Row-normalization yields an empirical conditional distribution:

(1) $$C_{ij}^{\left(s,e,d\right)}=\underset{s}{\text{Pr}}\left(y=j|x=i;e,d\right)\approx \frac{N_{ij}^{\left(s,e,d\right)}}{\sum_{j^{\prime }}N_{ij^{\prime }}^{\left(s,e,d\right)}}.$$

Let $C_{0}^{\left(s,e,d\right)}$ denote $C^{\left(s,e,d\right)}$ with its diagonal entries set to zero (i.e., excluding correct responses).

Each system is treated as a noisy communication channel from stimulus x to response y, and we infer a latent distortion matrix $\rho \in R_{\geq 0}^{K\times K}$ using maximum-a-posteriori (MAP) estimation. The likelihood is evaluated via the BA-optimal channel (Blahut, 1972; Arimoto, 1972) under ρ (with scale absorbed into ρ), following the RD fitting of Sims (2018) and the signature-extraction procedure introduced in Caglar et al. (2026) (BA frontier tracing, β/κ/AUC summarization), and adapted here to analyze directional asymmetry.

To trace the rate–distortion (RD) frontier, we scale ρ by an inverse-temperature parameter λ>0 and compute the corresponding optimal channel. Specifically, we solve for the RD-optimal channel $q_{\lambda }(y|x)$ using Blahut–Arimoto fixed-point updates. For a given distortion matrix ρ and λ, the updates proceed as:

$$q_{\lambda }\left(y|x\right)\propto p\left(y\right)\text{exp}(-\lambda \rho (x,y)),$$

$$p(y)=\sum_{x}p(x)q_{\lambda }(y|x).$$

These updates are iterated until convergence (with normalization over y implied).

We then trace the RD frontier over a log-spaced grid of λ values (e.g., $\lambda \in \left[10^{-1},10^{3}\right]$) and extract three compact RD signatures:

- Slope β: median finite-difference slope along the frontier, median{ΔR/ΔD},
- Curvature κ: variance of local finite-difference slopes along the frontier,
- Efficiency (AUC): trapezoidal area under the parametric curve R(D) over the swept λ range.

We use AUC rather than point-wise distance to the RD curve because it integrates efficiency across all operating points, providing a summary of the full frontier geometry rather than performance at a single compression level.

### 2.3 Asymmetry Metric Definitions

We define directional asymmetry as deviation from matrix symmetry in the row-normalized confusion matrix C. All metrics apply a threshold of $\varepsilon =10^{-12}$ to suppress numerical noise. Specifically:

$$n_{\text{pairs}}=\sum_{i<j}I\left[\left|C_{ij}-C_{ji}\right|>\varepsilon \right]$$

fpairs=npairs(K2)(fraction of asymmetric pairs)

$$\bar{\Delta }=E_{i<j}\left[|C_{ij}-C_{ji}|||C_{ij}-C_{ji}|>\varepsilon \right]$$

These metrics are used to quantify the extent and strength of asymmetry in the confusion structure. Analyses are performed on block-wise aggregates (defined by experiment × condition × model instance) to avoid pseudo-replication.

#### Frobenius Asymmetry.

Global asymmetry magnitude is quantified by the normalized Frobenius norm:

(2) $$A_{\text{F}}\left(C\right)=\frac{{\left\|C-C^{⊤}\right\|}_{\text{F}}}{\|C{\|}_{\text{F}}}.$$

#### Asymmetric Pair Counts and Directional Magnitude.

We summarize directional confusions using two complementary quantities: $f_{\text{pairs}}$ (breadth — how many class pairs exhibit asymmetry) and $\bar{\Delta }$ (strength — the mean magnitude of directional deviation among asymmetric pairs). The choice of ε and implementation details are provided in the Appendix. The primary asymmetry measure used for RD geometry linkage — the normalized off-diagonal Frobenius asymmetry $A_{\text{F}}^{\text{off}}$ — is defined in the following subsection, as it requires the diagonal-removed matrix $C_{0}$ introduced as part of the channel framework.

#### Group Comparisons.

We compare groups (e.g., Humans vs. CNNs) using Wilcoxon rank-sum tests for robustness to non-normality and Welch’s t-tests for effect size estimation. We report p-values corrected across planned comparisons using the Benjamini–Hochberg false discovery rate (BH–FDR) procedure. For each t-test, we compute 95% confidence intervals for the difference in means using the Welch–Satterthwaite approximation to the degrees of freedom.

### 2.4 Linking Asymmetry to RD Geometry

We test whether directional confusability covaries with RD behavior. Because the RD frontier is shaped by the full structure of the distortion matrix — including its asymmetric component — systems with different asymmetry organizations are expected to trace qualitatively different frontiers, even at matched accuracy levels. To quantify this, our primary asymmetry measure is the *normalized off-diagonal Frobenius asymmetry* computed from the row-normalized confusion probabilities. For each block, let C denote the row-normalized confusion matrix and let $C_{0}$ be C with its diagonal set to zero. We define

$$A_{\text{F}}^{\text{off}}(C)=\frac{{\left\|C_{0}-C_{0}^{⊤}\right\|}_{F}}{{\left\|C_{0}\right\|}_{F}}.$$

Channels with near-deterministic rows (e.g., collapsed responses) are flagged and excluded based on entropy and response dominance criteria computed from C (Appendix A1.1).

We then estimate asymmetry–RD relationships via:

- *(i) Rank correlations:* Spearman correlations within group between $A_{\text{F}}^{\text{off}}$ and each RD signature.
- *(ii) Block-controlled models:* Linear models with experiment/condition fixed effects and group-specific slopes.
- *(iii) Accuracy-controlled models:* Because asymmetry and RD geometry both covary with overall accuracy, we additionally test whether asymmetry–RD associations persist after controlling for accuracy within matched (experiment, condition) blocks, isolating the contribution of directional structure independent of performance level.

### 2.5 Mechanistic Simulation Linking Asymmetric Inductive Bias to RD Signatures

Our empirical results quantify asymmetry and RD geometry but do not expose the generative mechanisms behind their relationship. We therefore simulate systems with tunable asymmetric distortion structures to test interpretability and recoverability. Specifically, we predict that broad–weak and sink-like asymmetry organizations will produce opposite effects on RD geometry. Distributed asymmetries should expand the RD frontier by preserving information across many class distinctions, while concentrated sink-like asymmetries should collapse it by funneling probability mass into a few dominant responses — and that this dissociation should persist even after controlling for overall accuracy.

#### Simulation Setup.

We fix K=16 classes. Each replicate involves a ground-truth distortion matrix $\rho_{\text{true}}$, simulated confusion counts N, inferred distortion $\overset{ˆ}{\rho }$, and derived metrics. We construct $\rho_{\text{true}}$ as

$$\rho_{\text{true}}=\rho_{\text{sym}}+aA,\;\rho_{\text{sym}}=\rho_{\text{sym}}^{⊤},\;A=-A^{⊤},\;\rho_{ii}=0.$$

and evaluate two antisymmetry types: (i) *broad–weak* (dense skew-symmetric noise), and (ii) *sink-based* (targeted bias toward a small set of sink classes).

#### Channel Generation, Sampling, and RD Model Fitting.

Given $\rho_{\text{true}}$, a generation inverse temperature $\lambda_{\text{gen}}$, and a uniform stimulus prior p(x)=1/K, we generate channels via BA iterations and draw counts via

$$N_{i}.\sim \text{Multinomial}\left(N_{\text{per row}},q_{\lambda_{\text{gen}}}(\cdot |x=i)\right),$$

before recovering $\overset{ˆ}{\rho }$ by applying the same MAP RD pipeline used for empirical systems (see Sec. 2.2).

#### Simulation Grid and Diagnostics.

We compute RD signatures from both $\rho_{\text{true}}$ and $\overset{ˆ}{\rho }$, and compute asymmetry metrics from the corresponding induced channels/sampled confusions. Simulations are run over grids varying antisymmetry magnitude a, generation inverse temperature $\lambda_{\text{gen}}$, and per-class trial count $N_{\text{per row}}$, across both antisymmetry structures (broad–weak, sink) with multiple seeds. We reserve β for the empirical RD slope metric (generalization strength) and use λ to denote inverse-temperature parameters in Blahut–Arimoto and simulation generation. For pairwise breadth/strength summaries we threshold asymmetric pairs at $\varepsilon =10^{-6}$, and for each replicate we collect RD metrics, asymmetry scores, and recovery diagnostics (results reported in the Appendix). We also fit mixed-effects models and apply BH–FDR correction across trend tests. To validate our simulation-based inference, we computed secondary diagnostics, including Laplace-smoothened asymmetry as a sensitivity check, the operating point slope $s^{⋆}$ for both $\rho_{\text{true}}$ and $\overset{ˆ}{\rho }$, and the correlation between ground-truth and fitted distortion matrices for both their symmetric and antisymmetric components. Full results are reported in Appendix A3 (Tables 3–5).

## 3 Results

### Asymmetry magnitude and sparsity dissociate between humans and ANNs.

**Figure 1:**
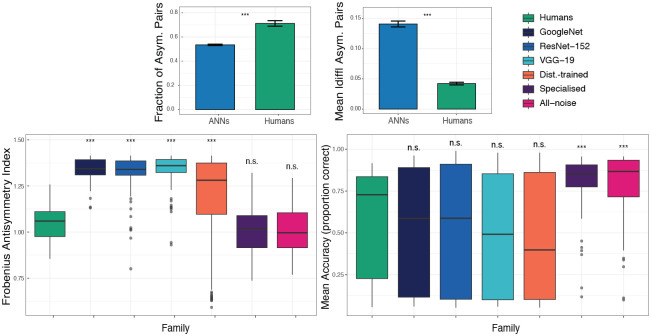
Asymmetry decomposes into *breadth* vs. *strength*, revealing a dissociation between humans and ANNs that is invisible to accuracy. **Top-left:** *Breadth* of directional structure quantified as the fraction of asymmetric class pairs ($f_{\text{asym}}$). **Top-right:** *Strength* of directional structure quantified as the conditional mean magnitude among asymmetric pairs. Error bars show s.e.m. across blocks; significance marks correspond to two-sided Wilcoxon rank-sum tests on block-wise summaries. **Bottom-left:** Global confusion-matrix asymmetry measured by the Frobenius index from row-normalized confusion matrices. Each point summarizes one block (unique experiment×condition×model), and boxes show the distribution across blocks. Significance is based on planned Wilcoxon comparisons of each group against humans with BH–FDR correction. **Bottom-right:** Mean classification accuracy (proportion correct) across the same blocks. Significance is based on planned Wilcoxon comparisons of each group against humans with BH–FDR correction. Groups that are significantly more asymmetric than humans are not significantly different in accuracy (n.s.), and vice versa, confirming a double dissociation between asymmetry structure and accuracy.

We quantified asymmetry using block-wise summaries (one value per unique *experiment*×*condition*×*model*; ANNs: n=1569 blocks; humans: n=81 blocks). ANNs exhibited larger *global* asymmetry than humans (see Fig. 1) as measured by the Frobenius index (mean±SE: ANNs 1.22 ± 0.0047 vs. humans 1.04 ± 0.0097; Wilcoxon rank-sum $p<2.2\times 10^{-16}$). Despite this larger global asymmetry, ANNs showed *sparser* directional structure. Humans had more asymmetric class pairs than ANNs (ANNs 64.2 ± 0.67 vs. humans 85.4 ± 2.76; Wilcoxon $p=2.24\times 10^{-11}$), equivalently a higher fraction of asymmetric pairs (ANNs 0.535 ± 0.0056 vs. humans 0.712 ± 0.023; Wilcoxon $p=2.24\times 10^{-11}$). This sparsity gap was even larger when restricting to baseline CNNs: only baseline ANNs 53.95 ± 1.45 vs. humans 85.4 ± 2.76 (Wilcoxon $p<2.2\times 10^{-16}$). Conversely, conditional on a pair being asymmetric, ANNs showed substantially larger per-pair directional deviations (conditional mean |Δ|: ANNs 0.141 ± 0.0049 vs. humans 0.0422 ± 0.0022; Wilcoxon $p=6.55\times 10^{-5}$), revealing a dissociation between *breadth* (more pairs in humans) and *strength* (larger deviations in ANNs).

Planned humans vs. model comparisons of Frobenius asymmetry (BH–FDR across groups) indicated that baseline CNNs and the Distortion-trained regimes remain significantly more asymmetric than humans, whereas the specialised and all-noise regimes were not significantly different from humans (Fig. 1), suggesting that robustness-oriented training can reduce global asymmetry toward the human range (see Table 1 for full test statistics and Table 2 for effect-size summaries). However, as we show below, this reduction in global asymmetry does not recover the human-like breadth–strength organization, indicating that scalar asymmetry metrics are insufficient proxies for representational alignment. This dissociation suggests that humans and ANNs impose qualitatively different priors under distribution shift: humans distribute errors broadly across the similarity space, while ANNs concentrate failures into a small number of dominant collapse directions. Importantly, this dissociation runs in both directions (Fig. 1, bottom-right). Groups that are significantly more asymmetric than humans (GoogleNet, ResNet-152, VGG-19, Distortion trained) show no significant difference from humans in accuracy, whereas groups that match humans on asymmetry (Specialised, All-noise) are significantly more accurate than humans. This double dissociation confirms that asymmetry structure and accuracy are genuinely independent and that directional confusion structure captures inductive bias information invisible to performance-based evaluation.

**Figure A1:**
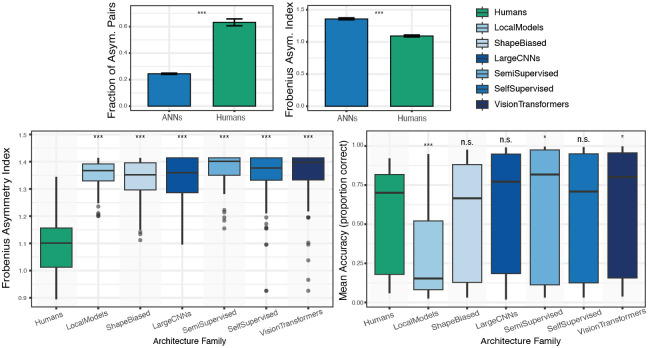
Asymmetry decomposes into *breadth* vs. *strength*, revealing a dissociation between humans and ANNs that is invisible to accuracy. **Top row:** Fraction of asymmetric class pairs (left) and Frobenius asymmetry index (right) comparing humans against all 15 modern model architectures. Error bars show s.e.m. and significance marks correspond to two-sided Wilcoxon rank-sum tests on block-wise summaries. **Bottom row:** Frobenius asymmetry index (left) and mean classification accuracy (right) by architecture family. Each point summarizes one block (unique experiment×condition×model). Boxes show the distribution across blocks. Significance marks are based on planned Wilcoxon comparisons of each family against humans with BH–FDR correction.

To assess whether the global asymmetry dissociation extends beyond classic CNN models, we replicated the Frobenius asymmetry and accuracy analysis using the same stimuli and experimental perturbations on modern models spanning 15 architectures including Vision Transformers, big transfer models, and more. All model families remained significantly more asymmetric than humans (all p < 0.001, BH-FDR; Fig. A1) regardless of accuracy level - including architectures that significantly outperform humans in accuracy — confirming that the asymmetry dissociation is not specific to the older CNN architectures evaluated in the main analysis and is not reducible to accuracy differences (see Appendix A2 for model descriptions and results). Subsequent analyses use the GEN repository data, which provides denser confusion matrices and directly matched human psychophysics under identical stimulus conditions needed for the analyses.

### 3.1 Asymmetry tracks RD efficiency and curvature beyond collapse artifacts

**Figure 2:**
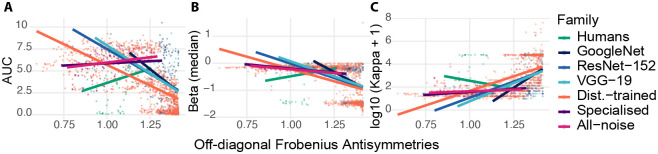
Directional confusion asymmetry covaries with rate–distortion (RD) signatures across humans and model families. Each point corresponds to one *block* (a unique experiment×condition×model instance) summarized by a row-normalized K×K confusion matrix. The x-axis reports *off-diagonal confusion asymmetry* $A_{\text{F}}^{\text{off}}$ in probability space, computed from the row-normalized confusion matrix with the diagonal removed (see Section 2.3). Thick lines show family-wise linear trends. **(A) Efficiency (AUC).** RD efficiency (area under the inferred RD curve) as a function of confusion asymmetry, illustrating how greater directional imbalance can coincide with reduced information–distortion efficiency in several ANN families, with a distinct profile for humans. **(B) Slope (β).** RD slope signature (median finite-difference slope ΔR/ΔD along the traced frontier) versus asymmetry, indicating how directional confusions track changes in the steepness of the information–error trade-off. **(C) Curvature (${\text{log}}_{10}(\kappa +1)$).** RD curvature proxy (log-transformed) versus asymmetry, highlighting regime-dependent coupling between directional structure and nonlinearity of the RD frontier.

Across systems and perturbation conditions, asymmetry in probability space was systematically related to the geometry of the inferred RD trade-off, but the direction and strength of the association depended on model family and training regime. Critically, several associations attenuated under accuracy control, indicating that naive correlations partly reflect shared performance dependence. The accuracy-matched analyses reveal where residual structure — the signature of inductive bias rather than performance level — genuinely links directional confusability to RD geometry. Throughout this section (Fig. 2), we quantify RD geometry using AUC (efficiency), β (global RD slope), and ${\text{log}}_{10}(\kappa +1)$ (curvature on a log scale), and we mark/exclude collapsed channels using the entropy/row-max criteria in Appendix A1.1.

#### Efficiency (AUC).

Greater asymmetry was associated with lower RD efficiency across most ANN families, but this coupling was largely driven by shared dependence on accuracy rather than residual directional structure — with one important exception in the Distortion-trained regime. Pooled rank correlations were strongly negative for Distortion-trained models (ρ=-0.73,n=1182) and also negative for the Baseline CNNs (GoogLeNet: ρ=-0.40; ResNet-152: ρ=-0.39; VGG-19: ρ=-0.43; each n≈80). A block-demeaned interaction model (demeaning within (experiment,condition) blocks) further indicated a substantially stronger within-block asymmetry–AUC dependence for Distortion-trained models than for humans ($\Delta \text{slope}=-7.89,p=1.27\times 10^{-7}$), with an additional negative interaction for Specialised models (Δslope=-4.77,p=0.014) and a directionally negative but only marginal effect for All-noise (Δslope=-3.55,p=0.055). However, because asymmetry is itself tightly coupled to performance accuracy, we additionally tested whether these patterns persist after accounting for accuracy differences within blocks. In an accuracy-controlled block-demeaned regression, the Distortion-trained models exhibited a robust *positive* conditional association between asymmetry and efficiency (accuracy-controlled within-block $\text{slope}=1.02\pm 0.12,t=8.74,p=6.0\times 10^{-18}$), while humans and baseline models showed no reliable accuracy-controlled slopes (all p≥0.14; humans: slope=-0.89,p=0.064). Thus, the negative pooled associations largely reflect shared dependence on accuracy, whereas the accuracy-matched analysis reveals regime-specific residual structure in how directional confusability relates to RD efficiency.

#### Slope (β).

A similar pattern held for RD slope: asymmetry and β were negatively correlated across most regimes, but these associations were largely mediated by accuracy, with accuracy-independent coupling emerging only in the Specialised regime. Specifically, Distortion-trained models showed a negative marginal association (ρ=-0.57,n=1182) and Baseline CNNs exhibited negative rank correlations as well (GoogLeNet: ρ=-0.33; ResNet-152: ρ=-0.46; VGG-19: ρ=-0.35). A block-demeaned interaction model indicated a steeper within-block dependence for Distortion-trained models than for humans (Δslope=-1.21,p=0.0085), with a weaker but statistically reliable negative interaction for Specialised models (Δslope=-1.22,p=0.0418), and no significant interaction for All-noise models (Δslope=-0.83,p=0.142). Importantly, these effects were not uniformly robust to accuracy control. In the accuracy-controlled block-demeaned analysis, Distortion-trained models no longer showed a reliable asymmetry–β relationship (slope=0.07,p=0.46), whereas Specialised models retained a significant negative association (slope=-0.79±0.39,t=-2.02,p=0.044). Baseline CNNs again showed no reliable accuracy-controlled effects (all p≥0.25). Together, these results indicate that apparent monotone associations between asymmetry and β can be driven by accuracy variation, with the clearest accuracy-independent coupling to β arising in the Specialised regime.

#### Curvature (κ).

Curvature showed the most accuracy-dependent pattern of the three RD signatures. Apparent positive associations with asymmetry in robustness-trained regimes disappeared entirely under accuracy control, suggesting that curvature primarily tracks performance rather than directional structure. Distortion-trained models showed a strong positive rank association (ρ=0.72,n=1182) and a significantly steeper within-block asymmetry–curvature dependence than Humans in the block-demeaned interaction model ($\Delta \text{slope}=3.96,p=1.97\times 10^{-5}$). Analogous positive interaction slopes were observed for All-noise (Δslope=2.56,p=0.0256) and Specialised (Δslope=3.40,p=0.0049) models, despite Specialised exhibiting an opposite pooled rank tendency (ρ=-0.31). However, this curvature effect is not robust to controlling for accuracy. In an accuracy-controlled within-block interaction model, accuracy was strongly predictive of curvature ($A_{\text{dm}}=-4.22,p=9.04\times 10^{-14}$), whereas neither the main within-block asymmetry term ($x_{\text{dm}}:p=0.153$) nor any asymmetry-by-group interaction was significant (Distortion-trained: p=0.450; Specialised: p=0.388; All-noise: p=0.871; baselines: all p≥0.251). Thus, the apparent asymmetry–curvature coupling in robustness-trained models is largely explained by shared variance with accuracy rather than an accuracy-independent link between directional confusability and RD curvature.

### 3.2 Mechanistic Simulation

We implemented the mechanistic simulation to ask the following targeted question: *When directional confusions increase, does the underlying rate–distortion (RD) geometry expand in the same way for different forms of asymmetry?* We compared two generators matched on the same control parameters (generalization regime and sample size) but differing in how directionality is organized. There was one *broad–weak* mechanism that distributes weak one-way biases across many class pairs, versus a second *sink-like* mechanism that concentrates probability mass into a small set of strong one-way errors. We report trends over all non-collapsed runs and use a strict-recovery filter only as a sensitivity check (see Appendix A3).

#### Directional structure is recoverable but identifiability is mechanism-dependent.

Across the full simulation grid (n=1800 runs), numerical collapse was rare (138*/*1800 = 7.7%), leaving n=1662 non-collapsed runs for primary analyses. A stricter reliability screen, requiring that the *recovered symmetric component* aligns with ground truth (correlation > 0.2), removed an additional 509*/*1662 = 30.6%, yielding n=1153 strictly-recovered runs (Appendix 3). Recovery was strongly *mechanism-dependent*, with broad–weak structure exceeding sinks in pass rate in every ($\lambda_{\text{gen}},N_{\text{per row}}$) slice and FDR significance in 10*/*15 slices (BH–FDR; Appendix Table 3). The largest gaps occurred at moderate-to-high generalization sharpness and intermediate-to-large sample sizes (maximum pass-rate gap 0.417; e.g. 0.867 vs 0.450), indicating that sink-like directional structure is intrinsically harder to identify under the same MAP pipeline. Consequently, we report all main trends on the *non-collapsed* set and use strict-recovery only as a sensitivity check (Appendix 3). This differential recoverability implies that our MAP pipeline may underestimate the prevalence of sink-like structure in empirical data. Results involving sink-like regimes should therefore be interpreted with this asymmetry in mind.

#### Broad–weak versus sinks produce opposite couplings between antisymmetry and RD geometry.

**Figure 3:**
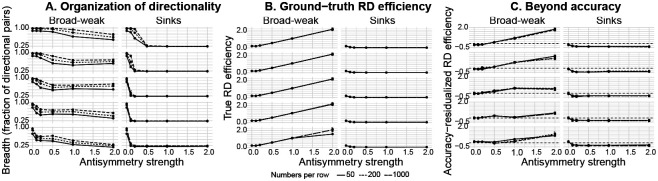
Mechanistic simulation: directional organization controls RD signatures. Columns compare antisymmetry generators (broad–weak vs. sinks); line types indicate the per-class trial budget $N_{\text{per row}}$. Facet rows correspond to the generation inverse-temperature values $\lambda_{\text{gen}}\in \{0.2,0.5,1,2,5\}$ (top to bottom). All panels show non-collapsed runs. **(A)** Breadth of directional structure (fraction of asymmetric class pairs, $f_{\text{pairs}}$) versus antisymmetry strength a. **(B)** Ground-truth RD efficiency (AUC of R(D) computed from $\rho_{\text{true}}$) versus a. **(C)** Beyond accuracy: within-slice residualized true RD efficiency versus a, where residuals remove the linear effect of mean diagonal probability within each fixed ($\lambda_{\text{gen}},N_{\text{per row}}$) slice. As a increases, the sink generator rapidly becomes sparse in directionality (A) and shows little-to-no increase in efficiency (B), leaving near-zero/negative accuracy-adjusted change (C), whereas the broad–weak generator maintains higher breadth (A) and exhibits a systematic efficiency increase that persists after accuracy residualization (B–C).

Consistent with our prediction, the same increase in antisymmetry strength produces opposite changes in RD geometry depending on whether directionality is distributed broadly or concentrated into sinks. In the broad–weak generator, increasing antisymmetry systematically *expands* ground-truth RD geometry, whereas in the sink-like generator it *collapses* ground-truth RD geometry toward a near-degenerate regime. This qualitative dissociation is visible in the true RD efficiency curves across regimes and remains consistent under strict-recovery filtering (Fig. 3; Appendix 4). This provides a concrete generative explanation for the empirical dissociation in which humans and ANNs can exhibit comparable global asymmetry magnitude yet occupy different regions of RD space.

#### The RD–asymmetry coupling is not reducible to overall performance.

A natural concern is that RD geometry might simply track overall performance. To test this, we removed the effect of an accuracy proxy (mean diagonal probability) *within each fixed* ($\lambda_{\text{gen}},N_{\text{per row}}$) operating slice and re-examined the dependence of RD efficiency on antisymmetry strength. This showed that the mechanism dissociation persisted. In the broad–weak generator, the accuracy-adjusted RD efficiency increases strongly with antisymmetry across every slice (typical slopes ≈ 0.87 to 1.03, all $p_{\text{FDR}}\leq 7.9\times 10^{-31}$), while in sinks, the corresponding slope is near-zero and slightly negative (≈ −0.046 to −0.064). The difference in slope between mechanisms is large and consistent across all 15 slices (interaction estimates ≈ −0.93 to −1.08, all $p_{\text{FDR}}\leq 5.0\times 10^{-29}$; Fig. 3; Appendix 3). Thus, the effect of directional structure on RD geometry cannot be explained away by accuracy.

#### The breadth–strength decomposition is predictive.

To connect mechanism to observable summaries, we decomposed directional structure into *breadth* (how many class pairs exhibit directionality) and *strength* (how large the directional deviation is among asymmetric pairs). After residualizing outcomes for accuracy *within* ($\lambda_{\text{gen}},N_{\text{per row}}$) slices, we asked which aspect of asymmetry explains residual variation in RD signatures (Appendix 4). For residual RD *efficiency* (AUC), the component model showed strong and slice-consistent effects. Breadth was typically negative (median coefficient −2.05, range [−5.40, 1.52]; significant in 11*/*15 slices, BH–FDR) and strength was also typically negative (median −4.28, range [−11.56, 2.45]; significant in 12*/*15 slices). Importantly, their interaction was typically positive (median +5.33, range [−4.65, 16.27]; significant in 5*/*15 slices), indicating that residual AUC depends on *how* directionality is distributed (broad-and-weak vs sparse-and-strong mixtures), not merely “more” or “less” asymmetry. In contrast, a one-number global magnitude model was weak for AUC residuals (Frobenius coefficient median −0.0047, range [−0.047, 0.206]; significant in only 3*/*15 slices). These results formalize the key point emerging from the empirical data, namely that *global asymmetry magnitude can obscure mechanistically meaningful organization*.

#### Slope and curvature behave differently from AUC: magnitude is sufficient.

The residualized RD slope magnitude and curvature show a complementary pattern. For slope magnitude, global magnitude is highly predictive across all regimes (Frobenius coefficient negative in 15*/*15 slices; median −0.144, range [−0.202, −0.063], BH–FDR), whereas breadth is weaker and less reliable (median −0.783, significant in 3*/*15 slices) and strength is more consistently negative (median −1.95, significant in 10*/*15 slices). For curvature (log-transformed), global magnitude is again uniformly predictive (Frobenius coefficient negative in 15*/*15 slices; median −0.201, range [−0.438, −0.089], BH–FDR), while breadth/strength terms are inconsistent. Thus, AUC seems to be the RD summary for which the *organization* of asymmetry (breadth vs strength) matters most, whereas slope and curvature primarily reflect overall directional magnitude (see Table 4).

#### Summary of mechanistic inference.

Together, the simulation supports a mechanistic interpretation aligned with the empirical results: (i) directional structure can be organized as broad–weak or sink-like, (ii) these regimes produce opposite RD-geometry consequences as antisymmetry increases, (iii) this coupling persists after controlling for accuracy, and (iv) the breadth–strength decomposition is essential for explaining RD efficiency differences that are invisible to a single global asymmetry magnitude.

## 4 Discussion

Human and ANN vision systems can achieve similar categorization accuracy under controlled image perturbations while relying on different inductive biases. Our findings show that directional confusions provide a compact behavioral signature of these biases, which are invisible to accuracy alone. Across matched human and model responses, we find a consistent dissociation in the *organization* of asymmetry. Humans exhibit broader but weaker directional structure, whereas ANNs exhibit sparser but stronger one-way collapses into dominant responses. Crucially, this difference is not captured by global asymmetry magnitude. Even though robustness-oriented training can reduce aggregate asymmetry toward the human range, it does not reliably recover the human-like breadth–strength profile, showing that two systems can match scalar metrics while failing for qualitatively different reasons. These differences reflect distinct priors about which features and prototypes are privileged under uncertainty.

By linking directional confusions to rate–distortion (RD) signatures inferred from confusion matrices, we test whether asymmetry *organization* predicts the *geometry* of the RD frontier. Empirically, breadth–strength structure was systematically reflected in RD signatures, as AUC (efficiency) and the shape descriptors β (slope) and κ (curvature) varied systematically with asymmetry organization, above and beyond global magnitude. In this effective-channel view, AUC summarizes overall information–error efficiency across operating points, whereas β and κ capture how sharply and how unevenly information must increase to avoid costly confusions. At the representational level, the breadth–strength dissociation we observe behaviorally is consistent with differences in the geometry of learned feature spaces. Broad–weak asymmetries, as seen in humans, are consistent with representational manifolds in which categories are arranged along graded similarity gradients (anisotropic but smoothly varying) such that many class pairs are weakly but meaningfully separated. Sink-like asymmetries, as seen in ANNs, are consistent with representations that collapse many inputs onto a small set of dominant attractor states, producing strong but sparse directional biases. This geometric interpretation connects our behavioral findings to neural manifolds, suggesting that directional confusion structure could serve as a behavioral probe of representational geometry without requiring direct access to internal activations.

Several asymmetry–RD associations nonetheless attenuate under accuracy control, indicating that naive correlations can partially reflect shared dependence on performance rather than structure alone. Our mechanistic simulations reinforce this point by showing that the same increase in directional asymmetry can produce *opposite* RD behaviors depending on how asymmetry is organized. In our simulations, broad–weak asymmetries shift the RD frontier toward higher efficiency, whereas sink-like asymmetries shift the frontier toward lower efficiency. These effects persist even under accuracy control in the simulation. Together, the simulations formalize a mechanistic link between the *organization* of directional confusions and the capacity–generalization trade-offs that shape behavior. This provides a mechanistic account of our second hypothesis, showing that the *same* asymmetry magnitude can induce different RD frontier geometry depending on whether asymmetries are broad–weak or sink-like. This is consistent with the broader pattern of RD geometry diagnostics documented across perturbation types and model families in Caglar et al. (2026), and extends that framework by showing that directional *organization* and not just magnitude is the key driver of RD frontier differences.

Even when trained for robustness or human-level accuracy under shift, models may distribute errors and representational resources differently than humans, yielding qualitatively different failure modes. Consequently, asymmetry structure, especially when linked to RD geometry, offers a principled and data-driven tool for probing those differences. Practically, this implies that matching human accuracy (or even matching aggregate asymmetry) is not sufficient to induce human-like robustness, since systems can achieve similar performance while concentrating failures into sink-like one-way collapses. This conclusion extends beyond the CNN architectures evaluated in the main analysis to a broad variety of modern model architectures as well (see Appendix A2, confirming that the observed dissociation is not model or architecture specific.

A natural target for human-aligned robustness is therefore not only reducing error, but redistributing error structure toward broader and weaker directional patterns. One concrete direction is to use the breadth–strength decomposition of asymmetry as a training signal. Penalizing sink-like collapse during training — for instance, by adding a regularization term that encourages directional errors to be distributed across many class pairs rather than concentrated into dominant responses — could push models toward the human-like regime without requiring matched accuracy. Such objectives could be computed directly from confusion matrices accumulated during training, making them practical for standard classification pipelines. Whether this redistribution of error structure would also improve robustness to distribution shift, as the human breadth–strength profile suggests, is an empirical question worth investigating directly.

Naturally, several open directions follow. First, moving from global summaries to class-level asymmetry can reveal which confusions drive the observed patterns, and whether models fail in the same directional subspaces as humans. Second, comparing antisymmetric components of the inferred distortion matrices may illuminate feature-level differences in model vs human inductive structure. Finally, while the global asymmetry dissociation replicates across modern architectures, extending the full breadth–strength and RD geometry analyses to these architectures (requiring denser matched confusion data) remains an important next step. More broadly, extending this framework to other modalities and task domains beyond visual categorization would test whether the breadth–strength dissociation reflects a general property of biological versus artificial inductive bias, or one specific to vision under distribution shift.

## Appendix

### A1 Statistical and Analytical Procedures

#### A1.1 Collapsed Channel Filtering

We exclude confusion matrices with collapsed responses based on diagnostics computed from the row-normalized conditional distribution C(y∣x) (i.e., each row of the confusion matrix normalized to sum to 1):

- Mean row entropy < 10^−3^, or
- Mean row-maximum probability > 0.999.

Only a small subset of system–condition matrices were excluded and this filtering had a negligible impact on the main trends.

#### A1.2 Asymmetry–RD Regression Models

We used two linear modeling approaches to assess how asymmetry covaries with RD signatures. First, models with fixed effects per (experiment, condition) block and group-specific slopes. Second, within-block demeaning of predictor and outcome variables, followed by regression with interaction terms.

### A2 Extension to Modern Architectures

#### A2.1 Model selection and data.

The main analyses use matched human psychophysics and ANN behavior from the GEN benchmark repository (Geirhos et al., 2018b), which provides ~83k human trials across 12 perturbation families at graded distortion levels for K=16 ImageNet-derived categories. Architecture choice in the main analysis was constrained by the requirement for matched human psychophysics data at sufficient trial density. Only models for which per-cell confusion counts are dense enough to reliably estimate directional asymmetry at the class-pair level are included. This limits the main analysis to three baseline CNNs (GoogLeNet, ResNet-152, VGG-19) and three robustness training regimes (Distortion-trained, Specialised, All-noise).

To assess whether the global asymmetry dissociation extends to modern architectures not included in the GEN repository, we additionally analyzed a broad selection of 15 architectures spanning five architecture families: LocalModels (BagNet-9, BagNet-33), ShapeBiased (ResNet50-SIN, ResNet50-SIN+IN), LargeCNNs (BiT-M R152×2, BiT-M R152×4), SemiSupervised (ResNeXt101-SWSL, EffNet-L2 NoisyStudent), SelfSupervised (SimCLR R50×1, SimCLR R50×4, MoCoV2, InfoMin), and VisionTransformers (CLIP, ViT-L/16, SWAG ViT-L/16). Model outputs were obtained from the ModelZoo repository Geirhos et al. (2021) using the same stimulus set, enabling direct comparison of asymmetry structure under identical perturbation conditions, with a second set of human data (~85k psychophysics trials).

#### A2.2 Analytical approach and sparsity caveat

Asymmetry metrics are computed identically to the main analysis (Section 2.3), using block-wise summaries (one value per unique experiment×condition×model) on row-normalized confusion matrices. Group comparisons use two-sided Wilcoxon rank-sum tests with BH–FDR correction across planned comparisons against humans.

An important limitation of the ModelZoo repository data is that confusion matrices are sparser than in the GEN repository, since these models were not evaluated with matched per-stimulus trial counts, resulting in fewer observations per confusion matrix cell. Then GEN repository confusion matrices contain on average 85 non-zero asymmetric class pairs out of 120 possible for humans (K=16; 71%) and 64 for ANNs (54%), providing sufficient density for the breadth–strength decomposition.

By contrast, the ModelZoo ANN confusion matrices contain on average only 29 pairs (24%), less than half the GEN ANN coverage despite identical K=16 categories and stimulus conditions. ModelZoo human data achieves comparable coverage to the GEN benchmark (76 pairs; 63%) confirming that the drop in pair coverage is specific to the ModelZoo ANN confusion matrices. This sparsity simultaneously deflates estimates of asymmetry breadth ($f_{\text{pairs}}$), because many off-diagonal cells are exactly zero, and inflates conditional magnitude (|Δ|), because only the largest asymmetries survive the sparsity filter. These two metrics should therefore be interpreted with caution for the ModelZoo data. The Frobenius asymmetry index, which aggregates over all off-diagonal entries and is substantially less sensitive to the proportion of zero cells, is robust to this issue and is therefore the primary metric reported here.

#### A2.3 Results

All 15 ModelZoo architecture families remain significantly more asymmetric than humans on the Frobenius index (all p<0.001, BH–FDR corrected; Fig. A1, bottom-left)), replicating the global asymmetry dissociation observed in the main analysis across a substantially broader range of architectures. This result holds regardless of training paradigm, including self-supervised (SimCLR, MoCoV2, InfoMin), semi-supervised (ResNeXt101-SWSL, EffNet-L2), shape-biased (ResNet50-SIN), and large-scale supervised models (BiT-M). Critically, it also holds for Vision Transformers and CLIP, which represent a qualitatively different architectural class from the CNNs evaluated in the main analysis.

The accuracy profile across architecture families is heterogeneous (Fig. A1, bottom-right). Local Models (BagNet-9, BagNet-33) are significantly less accurate than humans, while Vision Transformers (CLIP, ViT-L/16, SWAG ViT-L/16) are significantly more accurate. The majority of families are not significantly different from humans in accuracy. Yet all families are significantly more asymmetric than humans, confirming a double dissociation between asymmetry structure and accuracy that extends beyond the main analysis to modern architectures.

Subsequent analyses in the main paper only use the GEN data, which provides denser confusion matrices enabling reliable breadth–strength decomposition, and directly matched human psychophysics under identical stimulus conditions - both of which are necessary for the full RD geometry linkage and accuracy-controlled analyses reported in Sections 3.1 and 3.2.

### A3 Secondary diagnostics

#### A3.1 RD Signatures: Global vs. Local.

We summarize RD geometry from the inferred costs using two complementary notions of *slope*:

1. **Global RD slope and curvature (primary).** For each cost matrix ($\rho_{\text{true}}$ and ρ) we compute an RD curve by sweeping an inverse-temperature parameter λ over a fixed grid and recording (R(λ), D(λ)). We define the global slope signature β (defined as the median finite-difference derivative along the frontier, β=median{ΔR/ΔD}. We define curvature κ as the variance of these finite-difference slopes, and efficiency (AUC) as the trapezoidal area under the RD curve.
2. **Local operating-point slope (secondary).** We compute the empirical operating rate $R^{⋆}=I(X;Y)$ from the sampled confusion *counts* (using the empirical stimulus prior), then recover the local slope s by root-finding for the value of λ at which the optimal channel under cost ρ attains mutual information $R^{⋆}$.

### A3.2 Simulation Results

#### Recovery sensitivity and regime-wise pass-rate tests.

We report full recovery tables and perslice two-sample proportion tests (BH–FDR), confirming broad–weak > sinks pass rates in all 15 slices and FDR significance in 10*/*15. We additionally summarize how recovery rates vary with $\lambda_{\text{gen}}$ and $N_{\text{per row}}$ and emphasize that strict-recovery contrasts are conditional on identifiability, not unconditional mechanism differences.

#### Finite-sample error diagnostics for fitted versus true RD geometry.

We provide regime-wise error plots showing that sample size primarily stabilizes the recovered RD *geometry* summaries (AUC) and reduces dispersion, while local/global slope-related quantities can exhibit sporadic failures that are mechanism-specific. These diagnostics motivate using non-collapsed data for primary trends and strict recovery as a conservative sensitivity analysis.

#### Saturation behavior for sink-like organization.

We quantify the rapid transition of sink-like organization into a sparse, high-strength regime by estimating the knee point in breadth decline (and corresponding saturation in global magnitude), and we report these knee estimates by ($\lambda_{\text{gen}},N_{\text{per row}}$) slice. This analysis supports the interpretation that sink-like directionality quickly concentrates into a small set of one-way confusions and then becomes insensitive to further increases in antisymmetry strength.

#### Strict-recovery robustness of residual effects.

Finally, we repeat the key accuracy-residualized regressions under strict recovery and summarize sign/stability of the main effects in a compact table. This confirms that the qualitative pattern—AUC residuals requiring breadth–strength organization, and slope/curvature residuals tracking global magnitude—persists under conservative filtering.

**Table 1:** Block-wise asymmetry summary. One value per unique experiment×condition×model block. Values are mean±SE across blocks.

Metric	Blocks (A/H)	ANNs (mean±SE)	Humans (mean±SE)
Frobenius asymmetry index	1569 / 81	1.220 ± 0.0047	1.044 ± 0.0097
# asymmetric pairs ($n_{\text{pairs}}$), all ANNs	1569 / 81	64.16 ± 0.666	85.41 ± 2.76
# asymmetric pairs ($n_{\text{pairs}}$), baseline ANNs	243 / 81	53.95 ± 1.45	85.41 ± 2.76
Fraction of asymmetric pairs	1569 / 81	0.535 ± 0.0056	0.712 ± 0.023
Conditional mean magnitude (E[|Δ|||Δ|>0])	1569 / 81	0.1409 ± 0.0049	0.0422 ± 0.0022

**Table 2:** Asymmetry–RD associations by group. Spearman correlations between Frobenius asymmetry and RD metrics (AUC, $\beta_{\text{median}},{\text{log}}_{10}(\kappa +1)$), plus block-demeaned slope differences (Δslope) vs. humans. %Coll. reports the empirical collapse rate among behavioral confusion matrices (distinct from the 7.7% simulation collapse rate reported in Section 3.2). *p<0.05, **p<0.01, ***p<0.001.

(A) Rank correlations					
Group	n	%Coll.	$\rho_{\text{AUC}}$	$\rho_{\beta }$	$\rho_{\kappa }$
Humans	81	0.00	0.29	0.17	−0.22
GoogLeNet	81	0.00	−0.40	−0.33	0.43
ResNet-152	80	1.23	−0.39	−0.46	0.41
VGG-19	80	1.23	−0.43	−0.35	0.48
Distortion-trained	1182	3.11	−0.73	−0.57	0.72
Specialised	53	0.00	0.37	0.18	−0.31
All-noise	53	0.00	0.32	0.22	−0.36

(B) 𝚫slope vs. humans			
Group	AUC	β	κ
Humans		0 (ref.)	
GoogLeNet	2.67 (p=.34)	0.91 (p=.29)	−0.87 (p=.62)
ResNet-152	−0.69 (p=.74)	0.36 (p=.58)	−0.48 (p=.71)
VGG-19	−1.36 (p=.57)	−0.35 (p=.63)	0.61 (p=.68)
Distortion-trained	−**7.89** ($p=1.3\times 10^{-7}$)***	−**1.21** (p=.0085)**	**3.96** ($p=2\times 10^{-5}$)***
Specialised	−**4.77** (p=.014)*	−**1.22** (p=.042)*	**3.40** (p=.0049)**
All-noise	−3.55 (p=.055)	−0.83 (p=.14)	**2.56** (p=.026)*

**Table 3:** Recovery sensitivity. A run is counted as recovered if the correlation between recovered and true symmetric confusions exceeds 0.2. The table reports recovery fractions within each ($\lambda_{\text{gen}},N_{\text{per row}}$) slice and BH–FDR adjusted p-values for differences in recovery rates (two-sample proportion tests).

$\lambda_{\text{gen}}$	$N_{\text{per row}}$	Frac. recovered (broad–weak)	Frac. recovered (sinks)	𝚫 frac. (broad–weak − sinks)	$p_{FDR}$
0.2	50	0.567	0.433	0.133	0.262
0.2	200	0.700	0.583	0.117	0.343
0.2	1000	0.733	0.600	0.133	0.262
0.5	50	0.783	0.536	0.247	0.0267
0.5	200	0.817	0.604	0.213	0.0576
0.5	1000	0.800	0.607	0.193	0.0718
1	50	0.817	0.583	0.233	0.0419
1	200	0.833	0.583	0.250	0.0267
1	1000	0.850	0.617	0.233	0.0419
2	50	0.833	0.550	0.283	0.0137
2	200	0.883	0.600	0.283	0.0137
2	1000	0.900	0.617	0.283	0.0137
5	50	0.750	0.467	0.283	0.0137
5	200	0.833	0.533	0.300	0.00864
5	1000	0.850	0.550	0.300	0.00864

**Table 4:** Residual regression summary. Within each ($\lambda_{\text{gen}},N_{\text{per row}}$) slice, we first residualize each RD signature by mean diagonal probability (accuracy proxy), then regress the residual on either (i) breadth/strength terms or (ii) a global asymmetry magnitude term. “Structure offset” refers to contrast between sink-like and broad–weak configurations. Coefficients are summarized across slices; significance counts use BH–FDR within-slice tests.

Outcome	Predictor set	Term	Median	Min	Max	Sig. slices	Slices
AUC	components	Breadth	−2.05	−5.40	1.52	11	15
AUC	components	Strength	−4.28	−11.56	2.45	12	15
AUC	components	Breadth × Strength	5.33	−4.65	16.27	5	15
AUC	components	Structure offset	−0.447	−0.657	−0.162	15	15
AUC	magnitude	Global asymmetry mag.	−0.0047	−0.0470	0.206	3	15
AUC	magnitude	Structure offset	−0.482	−0.691	−0.171	15	15
Global slope	components	Breadth	−0.78	−2.12	0.37	3	15
Global slope	components	Strength	−1.95	−3.70	−0.40	10	15
Global slope	components	Breadth × Strength	−5.02	−9.96	5.73	7	15
Global slope	components	Structure offset	−0.188	−0.357	0.066	9	15
Global slope	magnitude	Global asymmetry mag.	−0.144	−0.202	−0.063	15	15
Global slope	magnitude	Structure offset	−0.243	−0.386	−0.081	15	15
Curvature	components	Breadth	−0.048	−0.167	0.129	3	15
Curvature	components	Strength	−0.165	−0.540	0.084	6	15
Curvature	components	Breadth × Strength	0.066	−1.030	1.250	2	15
Curvature	components	Structure offset	0.058	−0.011	0.124	7	15
Curvature	magnitude	Global asymmetry mag.	−0.201	−0.438	−0.089	15	15
Curvature	magnitude	Structure offset	0.060	−0.009	0.126	8	15

**Table 5:** Mixed-effects ANOVA key terms. Selected fixed-effect tests from replicate-level mixed models $Y\sim \text{structure}\times a\times \lambda_{\text{gen}}\times {\text{log}}_{10}N_{\text{per row}}+(1|\text{cell})$.

Outcome	Term	F	p
auc_true	structure	10.16	0.00146
auc_true	a_scale	1176	2.2e-16
auc_true	structure:a_scale	877.1	2.2e-16
beta_median_true_pos	structure	24.41	7.8e-07
beta_median_true_pos	a_scale	827.7	2.2e-16
beta_median_true_pos	structure:a_scale	613.4	2.2e-16
kappa_true	structure	5.34	0.0209
kappa_true	a_scale	964.7	2.2e-16
kappa_true	structure:a_scale	701.8	2.2e-16

## References

[R1] Arimoto Suguru. An algorithm for computing the capacity of arbitrary discrete memoryless channels. IEEE Transactions on Information Theory, 18(1):14–20, 1972.

[R2] Attarian Maria, Roads Brett D, and Mozer Michael C. Transforming neural network visual representations to predict human judgments of similarity. arXiv preprint arXiv:2010.06512, 2020.

[R3] Blahut R.. Computation of channel capacity and rate-distortion functions. IEEE Transactions on Information Theory, 18(4):460–473, 1972. doi: 10.1109/TIT.1972.1054855.

[R4] Caglar Leyla Roksan, Mediano Pedro AM, and Lin Baihan. Rate-distortion signatures of generalization and information trade-offs. arXiv preprint arXiv:2603.01568, 2026.

[R5] D’Amato Leo, Lancia Gian Luca, and Pezzulo Giovanni. The geometry of efficient codes: How rate-distortion trade-offs distort the latent representations of generative models. PLOS Computational Biology, 21(5):e1012952, 2025. 40354307 10.1371/journal.pcbi.1012952 PMC12068621

[R6] Geirhos Robert, Rubisch Patricia, Michaelis Claudio, Bethge Matthias, Wichmann Felix A, and Brendel Wieland. Imagenet-trained cnns are biased towards texture; increasing shape bias improves accuracy and robustness. In International conference on learning representations, 2018a.

[R7] Geirhos Robert, Temme Carlos RM, Rauber Jonas, Schütt Heiko H, Bethge Matthias, and Wichmann Felix A. Generalisation in humans and deep neural networks. Advances in neural information processing systems, 31, 2018b.

[R8] Geirhos Robert, Jacobsen Jörn-Henrik, Michaelis Claudio, Zemel Richard, Brendel Wieland, Bethge Matthias, and Wichmann Felix A. Shortcut learning in deep neural networks. Nature Machine Intelligence, 2(11):665–673, 2020.

[R9] Geirhos Robert, Narayanappa Kantharaju, Mitzkus Benjamin, Thieringer Tizian, Bethge Matthias, Felix A Wichmann, and Wieland Brendel. Partial success in closing the gap between human and machine vision. Advances in Neural Information Processing Systems, 34:23885–23899, 2021.

[R10] Getty David J, Swets John A, Swets Joel B, and Green David M. On the prediction of confusion matrices from similarity judgments. Perception & Psychophysics, 26(1):1–19, 1979.

[R11] Gupta Shashi Kant, Zhang Mengmi, Wu Chia-Chien, Wolfe Jeremy, and Kreiman Gabriel. Visual search asymmetry: Deep nets and humans share similar inherent biases. Advances in neural information processing systems, 34:6946–6959, 2021. 36062138 PMC9436507

[R12] Ilyas Andrew, Santurkar Shibani, Tsipras Dimitris, Engstrom Logan, Tran Brandon, and Madry Aleksander. Adversarial examples are not bugs, they are features. Advances in neural information processing systems, 32, 2019.

[R13] Jakob Anthony MV and Gershman Samuel J. Rate-distortion theory of neural coding and its implications for working memory. Elife, 12:e79450, 2023. 37435811 10.7554/eLife.79450 PMC10353860

[R14] Kahana Michael J and Sekuler Robert. Recognizing spatial patterns: A noisy exemplar approach. Vision research, 42(18):2177–2192, 2002. 12207978 10.1016/s0042-6989(02)00118-9

[R15] Kemp Charles and Tenenbaum Joshua B. The discovery of structural form. Proceedings of the National Academy of Sciences, 105(31):10687–10692, 2008. 10.1073/pnas.0802631105 PMC2492756 18669663

[R16] Liu Minghao, Wei Jiaheng, Liu Yang, and Davis James. Human and ai perceptual differences in image classification errors. In Proceedings of the AAAI Conference on Artificial Intelligence, volume 39, pp. 14318–14326, 2025.

[R17] Miller John P, Taori Rohan, Raghunathan Aditi, Sagawa Shiori, Koh Pang Wei, Shankar Vaishaal, Liang Percy, Carmon Yair, and Schmidt Ludwig. Accuracy on the line: on the strong correlation between out-of-distribution and in-distribution generalization. In International conference on machine learning, pp. 7721–7735. PMLR, 2021.

[R18] Nosofsky Robert M. Attention, similarity, and the identification–categorization relationship. Journal of experimental psychology: General, 115(1):39, 1986. 2937873 10.1037//0096-3445.115.1.39

[R19] Nosofsky Robert M. Stimulus bias, asymmetric similarity, and classification. Cognitive Psychology, 23(1):94–140, 1991.

[R20] Papyan Vardan, Han XY, and Donoho David L. Prevalence of neural collapse during the terminal phase of deep learning training. Proceedings of the National Academy of Sciences, 117(40): 24652–24663, 2020. 10.1073/pnas.2015509117 PMC7547234 32958680

[R21] Recht Benjamin, Roelofs Rebecca, Schmidt Ludwig, and Shankar Vaishaal. Do imagenet classifiers generalize to imagenet? In International conference on machine learning, pp. 5389–5400. PMLR, 2019.

[R22] Rosch Eleanor. Cognitive representations of semantic categories. Journal of experimental psychology: General, 104(3):192, 1975.

[R23] Rosch Eleanor and Mervis Carolyn B. Family resemblances: Studies in the internal structure of categories. Cognitive psychology, 7(4):573–605, 1975.

[R24] Shah Harshay, Tamuly Kaustav, Raghunathan Aditi, Jain Prateek, and Netrapalli Praneeth. The pitfalls of simplicity bias in neural networks. Advances in Neural Information Processing Systems, 33:9573–9585, 2020.

[R25] Shankar Vaishaal, Roelofs Rebecca, Mania Horia, Fang Alex, Recht Benjamin, and Schmidt Ludwig. Evaluating machine accuracy on imagenet. In International Conference on Machine Learning, pp. 8634–8644. PMLR, 2020.

[R26] Shannon Claude E. A mathematical theory of communication. The Bell system technical journal, 27(3):379–423, 1948.

[R27] Shepard Roger N. Attention and the metric structure of the stimulus space. Journal of mathematical psychology, 1(1):54–87, 1964.

[R28] Shepard Roger N. Toward a universal law of generalization for psychological science. Science, 237 (4820):1317–1323, 1987. 3629243 10.1126/science.3629243

[R29] Sims Chris R. Efficient coding explains the universal law of generalization in human perception. Science, 360(6389):652–656, 2018. 29748284 10.1126/science.aaq1118

[R30] Sloman Steven A. Categorical inference is not a tree: The myth of inheritance hierarchies. Cognitive Psychology, 35(1):1–33, 1998. 9520316 10.1006/cogp.1997.0672

[R31] Sorscher Ben, Ganguli Surya, and Sompolinsky Haim. Neural representational geometry underlies few-shot concept learning. Proceedings of the National Academy of Sciences, 119(43): e2200800119, 2022. 10.1073/pnas.2200800119 PMC9618072 36251997

[R32] Tversky Amos. Features of similarity. Psychological review, 84(4):327, 1977.

[R33] Tversky Amos and Gati Itamar. Similarity, separability, and the triangle inequality. Psychological review, 89(2):123, 1982. 7089125

[R34] Wei Xue-Xin and Woodford Michael. Representational geometry explains puzzling error distributions in behavioral tasks. Proceedings of the National Academy of Sciences, 122(4):e2407540122, 2025. 10.1073/pnas.2407540122 PMC11789072 39854237

